# Molecular Detection of *Leishmania major* in *Hemiechinus auritus*: A Potential Reservoir of Zoonotic Cutaneous Leishmaniasis in Damghan, Iran

**Published:** 2019-09-30

**Authors:** Behrad Pourmohammadi, Sadegh Mohammadi-Azni

**Affiliations:** 1 Department of Parasitology, School of Medicine, Semnan University of Medical Sciences, Semnan, Iran; 2 Department of Health Education and Promotion, School of Health, Semnan University of Medical Sciences, Semnan, Iran; 3 Damghan Health Center, Semnan University of Medical Sciences, Semnan, Iran

**Keywords:** *Hemiechinus auritus*, Potential reservoir, *Leishmania major*, Semi-nested PCR, Iran

## Abstract

**Background:**

Zoonotic cutaneous leishmaniasis caused by *Leishmania major* is endemic in 17 of 31 Iranian provinces. Various species of rodents have been introduced as the main reservoirs of the disease. This study was conducted to determine the natural infection of hedgehogs with *Leishmania* spp. in an endemic area of the disease, northern Iran.

**Methods:**

Fifteen long-eared hedgehogs were captured alive during 18 months study period, from Apr 2015 to Sep 2016, in Damghan City, Semnan Province, Iran. The animals were identified using apparent characteristics and to determine the *Leishmania* infection, impression smears were prepared from their ear lobes, hind feet, livers, and spleens. Microscopic examination and semi-nested PCR were applied to determine the infection and to identify the parasites species respectively.

**Results:**

All examined animals were identified as *Hemiechinus auritus* (Family: Erinaceidae). In microscopic examination, 8 (53.3%) samples were shown to be infected with *Leishmania* parasites. The higher and lower rate of the infection was observed in the ears as well as the feet and in the liver specimens, 53.3%, and 33.3% respectively. Forty percent (6/ 15) of the samples were molecularly positive and all were identified as *L. major* parasites. All the examined animals in autumn and 50% of them in summer were shown to be infected with *Leishmania* parasites.

**Conclusion:**

This study demonstrated the natural infection of *H. auritus* with *L. major* for the first time in Damghan City and introduced these mammals as new potential reservoirs of ZCL in the study area.

## Introduction

Leishmanioses, a significant group of protozoan parasitic diseases caused by around 20 of 30 obligate intracellular parasite species of genus *Leishmania* in humans, are endemic and occur in more than 100 countries of four continents of the world including; Asia, Africa, Europe, and America. The disease represents four types of clinical manifestations from the self-healing cutaneous to the life-threatening, visceral form, if untreated (1–3). So far, more than 50 species of genus *Leishmania* have been identified, of which 31 species can infect mammals and 20 species can cause the various types of leishmaniasis in humans with either zoonotic or anthroponotic origins (1, 4). No more than 70 species of approximately 900 sand fly species have been implicated in the transmission of Leishmaniasis (5).

Leishmaniasis is still one of the most neglected diseases in the world which affecting largely the poorest of the poor, mainly in developing countries (3). Overall, 350 million people are at risk of all types of disease and an estimated 1.6 million cases occur annually all over the world. There is also an estimation of 0.2–0.4 million cases of Visceral Leishmaniasis (VL) and 0.7–1.2 million cases of cutaneous leishmaniasis (CL) in the endemic areas of the world (6).

Cutaneous leishmaniasis is an endemic disease in 88 countries worldwide (7). Approximately, 90% of CL cases have been reported from Iran, Algeria, Afghanistan, Saudi Arabia, Syria, Brazil and Peru (8). Iran is one of the ten countries that around 70–75% of new CL cases occur in it (6).

Cutaneous leishmaniasis (CL), is one of the two clinical forms of the disease in Iran, is more prevalent with a prevalence rate of 1.8–37.9% and around 30000 new cases annually (9). Globally, it is estimated to be the ninth cause of human infectious diseases' burden (10). The zoonotic form of the disease (ZCL) due to *L. major* is the most prevalent type of the CL mainly in rural areas of 17 out of 31 provinces of the country (11, 12). According to the geographical distribution and various types of reservoirs and vectors of ZCL, three epidemiological types of the disease have been classified in Iran (13).

Various species of rodents including *Rhombomys opimus*, *Nesokia indica*, *Meriones libycus*, *Tatera indica*, *M. hurrianae*, *Rattus norvegicus*, *M. persicus*, and *Mus musculus* have been identified as reservoir and or potential reservoir hosts of the parasite in the country (11, 14–19). Furthermore, the parasite species have recently been isolated and reported in gorillas (20) and hedgehogs in several parts of the world (21–24). *Phlebotomus papatasi*, the most prevalent species of *Phlebotomus* genus, is the predominant and the only proven vector of ZCL (13, 25).

Hedgehogs are distributed in various parts of Iran. These mammals are found in the wild and in a lower amount of pet animals. Despite wide distribution in Iran, these animal species have not been studied enough about their parasites (26). Hedgehogs are omnivorous. They feed mostly on invertebrates such as slugs, beetles, caterpillars, earthworms, and some other insects. Furthermore, grass snakes, frogs, vipers, fish, young birds, birds' eggs and small rodents can be fed by those small mammals (27).

The long-eared hedgehog lives in burrows that it makes or finds. It is recognized by its long ears. They inhabit a few various types of dry steppes, deserts and semi-deserts. They often inhabit in oasis and around human settlements. The long-eared hedgehogs live in burrows that they dig under bushes with a length of 45cm long with one opening. They may live in abandoned burrows of other small mammals. They are nocturnal solitary hedgehogs. During the day, they are found resting under rocks, hollows or rock piles (28).

In a study to determine the reservoirs and vectors of *Leishmania* parasites in Damghan City, an endemic focus of ZCL in Iran, we accidentally encountered with a hedgehog captured in wire live trap set out for rodents in the southern part of the city, where the rodents infection with *L. major* parasites have been proven more recently (11). The existence of some recent reports about the infection of the hedgehogs with *Leishmania* parasites in other parts of the world drew our interest in assessing *Leishmania* spp. in these animals.

Therefore, the present study was conducted to investigate the possible leishmania infection of these small mammals in the city in an eighteen month period, from Apr 2015 to Sep 2016.

## Materials and Methods

### Study area and period

The present study was carried out during 4 Apr 2015 to 22 Sep 2016 in Damghan, a well-known endemic region of ZCL in Iran. Zoonotic Cutaneous Leishmaniasis have been reported in an epidemic form in Damghan district (at about 36°10′ N, 54°20′ E) with around 1000 cases in 1999. Since 1999 until now the disease remains in endemic form with around 100 cases every year (29, 30). Damghan is a city situated between Semnan and Shahroud cities of Semnan Province with an altitude of 1170m and arid climate. The temperature and annual rainfall averages of the city are about 16.3 °C and 120mm respectively. The city is bounded on the plain areas in the south with 23.5 °C and the mountainous areas in the north with 9.8 °C average temperature. However, it is classified as a desert region with an arid climate (31, 32).

### Animal capturing

**Fig. 1.**
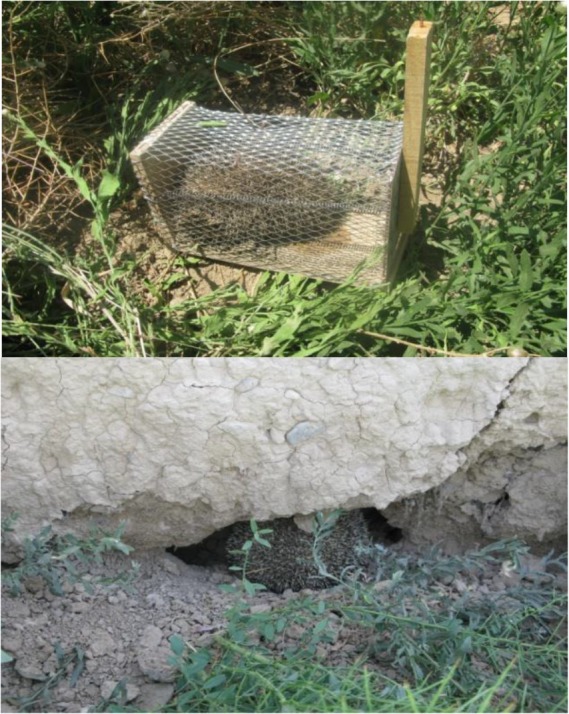
The hedgehog captured in wire trap set up at rodent’s burrow entrance and the animal found in a resort in the radix of an ancient wall surrounding the city

The first animal was captured in a wire live trap (Sherman) laid at the entrance of the rodent burrow in farmland of wheat close to houses (Fig. 1) and the rest of the animals were found in their daily resorts, holes in the old brick walls, and were caught by hand. The captured animals were put in a carton and were transferred to the laboratory.

The ethical code (IR.SEMUMS.REC.1397. 136) was obtained from the Ethics Committee of Semnan University of Medical Sciences.

### Measurements and sampling

After transferring to the laboratory and taking photographs, the animals were slightly anesthetized using chloroform for recording the apparent characteristics; sex, probable ulcers in each part of the body, and external measures of the ear, the hind foot, the head, the body and the tail of each animal. Impression smears were made from the hindfoot and the ear lobe on clean glass slides. Then, the sampling of spleen and liver were done after euthanizing the animals following deep anesthesia and autopsy. The animals were identified according to morphologic characteristics and using valid identification key (33).

### Microscopic investigation

All in absolute methanol fixed smears were stained using 5% Giemsa stain for 25min and were carefully examined under a light microscope at 1000× magnification. At least two slides of each sampled organs (ear lobe, hind foot, spleen, and liver) were checked to make the final decision about parasitological results.

### Molecular identification

For parasite species identification, the ears and feet smears of all fifteen samples were checked by a sensitive molecular technique, Semi-nested PCR assay.

### DNA extraction

The dry Giemsa-stained smears of the ears and feet of each animal were scratched off the slides using lysis buffer and *Leishmania* parasites total DNA was extracted by the YTA Genomic DNA Extraction Mini Kit (YEKTA Tajhiz Azma, Iran) following the manufacturer's training.

### Semi-Nested PCR amplification

The Semi-Nested PCR was performed, as formerly described and applied by other researchers (34–36), for detection of Kinetoplast DNA (kDNA) of *L. major* in the specimens prepared from ear lobes and hind feet of the hedgehogs. The applied primers were the forward LINR4 (5′- GGG GTT GGT GTA AAA TAG GG-3′), the first-round reverse LIN17 (5′- TTT GAA CGG GAT TTC TG-3′), and the second-round reverse LIN19 (5′-CAG AAC GCC CCT ACC CG-3′). In the first-round amplification reaction totally 10μl mixture containing 1μM 10X Buffer, 200μM of each dNTP, 1.5mM MgCI2, 1μM primer LINR4, 0.2μM primer LIN17, 1 unit Taq polymerase (CinnaGen, Tehran, Iran) and 1.5μl of target kDNA from each hedgehog’s specimens were prepared. The mixture was over-laid with mineral oil and then incubated in a CG1-96 thermocycler (Corbett Research, Sydney, Australia) at 94 °C for 5min succeeded by 17 cycles, each consisting of 30sec at 94 °C, 30 sec at 52 °C and 30sec at 72 °C. After the last cycle, the extension was continued for a further 10min then held at 4 °C. The second-round amplification was accomplished following the addition of 90μl of buffer comprising MgCl2, dNTPs and Taq polymerase, as described for the first round, and primer LINl9 (final concentration 1μM) for 33 cycles (94 °C for 30sec, 58 °C for 30sec and 72 °C for 1min). kDNA of reference strain of *L. major* (MHOM/IR/54/LV39) and distilled water were used as positive and negative controls respectively. 15μl of each PCR products were resolved in 5μl loading buffer and then electrophoresed in a 1.5% agarose gel in TBE buffer containing 0.75% ethidium bromide and visualized under ultraviolet transilluminator. *Leishmania* parasites were identified by comparison of PCR products of specimens with the reference strains and molecular weight markers.

## Results

### Animal measurements and descriptions

**Fig. 2.**
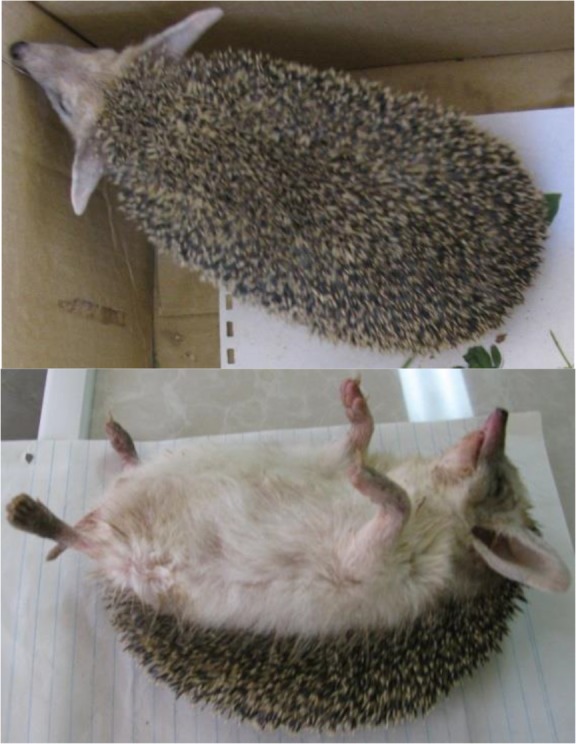
The examined hedgehogs in dorsal and ventrolateral view

During an 18-month period of the study, 4 Apr 2015 to 22 Sep 2016, 15 (8 male and 7 female) porcupines were caught from different locations in the city. The samples were caught in three seasons of the year. Ten of 15 examined animals were captured in the summer. None of the animals were caught in winter. All animals were morphologically identified as long-eared hedgehog, *Hemiechinus auritus* (Gmelin, 1770) belong into Family; Erinaceidae (33). The animals' dorsal spines were white on the peak with darker banding inferior. They had a light-colored underside along with long and highly visible ears (length, 41– 49mm) on the head with whitish hairs on the tips of them. The tops and heels of their feet (hind feet length, 37–45mm) were covered with hair but the soles were bare (Fig. 2). The length of the heads, the bodies, the tails and the ears were 50–65mm, 205–230mm, 21–26mm and 41–50 mm respectively (Table 1). None of the animals had any skin ulcers.

**Table 1.** Morphologic characteristics, measurements, parasitological and molecular results of the hedgehogs captured from Damghan City, Semnan Province, Iran, 2015 to 2016

**Animals**	**G**	**CD**	**Measurements (mm)**					**Parasitological result**				**Molecular result**

			**H**	**F**	**B**	**T**	**E**	**E**	**L**	**S**	**F**	
**H_1_**	F	22 May 2015	63	44	225	24	47	+	+	-	+	*L. major*
**H_2_**	M	28 May 2015	59	43	213	24	49	+	-	+	+	-
**H_3_**	M	25 Jun 2015	65	45	222	25	50	-	-	-	-	-
**H_4_**	F	3 Jul 2015	64	41	230	26	45	-	-	-	-	-
**H_5_**	M	4 Sep 2015	51	40	212	23	41	+	-	+	+	*L. major*
**H_6_**	F	14 Sep 2015	50	38	205	21	46	+	+	+	+	*L. major*
**H_7_**	M	27 Sep 2015	62	42	225	25	45	-	-	-	-	-
**H_8_**	M	1 Nov 2015	53	40	215	23	43	-	-	-	-	-
**H_9_**	F	3 Dec 2015	53	37	210	23	42	+	+	+	+	*L. major*
**H_10_**	F	1 Jul 2016	60	42	224	24	44	-	-	-	-	*-*
**H_11_**	M	8 Jul 2016	52	41	215	22	42	+	-	+	+	*-*
**H_12_**	M	22 Jul 2016	54	39	217	23	43	-	-	-	-	*L. major*
**H_13_**	F	5 Aug 2016	62	41	224	25	47	-	-	-	-	*-*
**H_14_**	M	19 Aug 2016	57	42	218	24	48	+	+	-	+	*-*
**H_15_**	F	9 Sep 2016	61	43	225	25	50	+	+	+	+	*L. major*
**Total**	15							8	5	6	8	6 (40%)
								8 (53.3%)				

**Abbreviations**: **G**= Gender, **CD**= Capture Date, **H**= Head, **F**= Foot, **B**= Body, **T**= Tail, **E**= Ear, **L**= Liver, **S**= Spleen

### Microscopic and molecular investigation

**Fig. 3.**
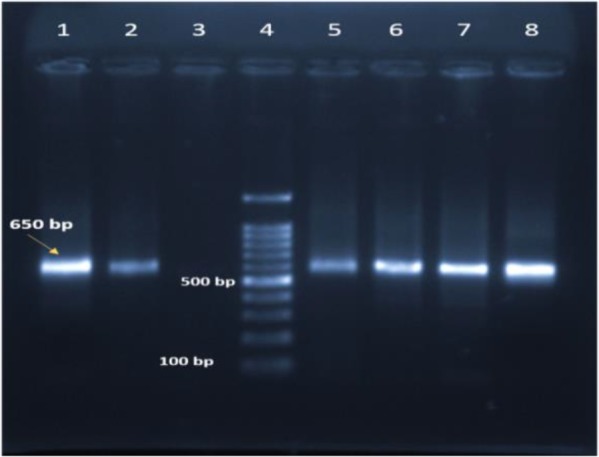
The PCR-based products' gel electrophoresis of the samples prepared from the *Hemiechinus auritus* samples captured from Damghan City. The bands correspond to Reference strain of *Leishmania major* (Lane 1), Hind feet smears (Lanes 2 and 5), Ear lobes samples (Lanes 6, 7 and 8), Ladder marker (Lane 4) and Negative control (Lane 3)

In the microscopic examination of the prepared smears from four organs (ear lobes, hind feet, liver, and spleen) of each 15 animals, eight (53.3%) of samples were shown to be infected with *Leishmania* parasites. The higher rates of infection were seen in the samples of ears the same as feet with 53.3% for each. The lower rates of infections (33.3%) were observed in liver specimens (Table 1). All the captured and the examined animals in autumn (3/3, 100%) and 50% (5/10) of the examined animals in summer were shown to be infected with *Leishmania* parasites. In the molecular assay, six of the specimens (40%) were identified as *L. major* with sharp bands (Fig. 3). Two of the parasitological positive samples were negative in the molecular assay.

## Discussion

Four species of hedgehogs including *H. auritus* (Long-eared hedgehog), *P. hypomelas* (Brandt's hedgehog), *P. aethiopicus* (Desert hedgehog), and *E. concolar* (European hedgehog) have been identified and reported in different parts of Iran (26, 37, 38).

All the captured animals in the present study were belonging to *Hemiechinus auritus* (Long-eared hedgehog). These spiny-coated small mammals are widely distributed throughout the country particularly in provinces surrounding the studied area, Damghan City, Semnan Province. They have been reported from Kurdistan, Khuzestan, Golestan, Khorasan, Qazvin, Tehran, Sistan and Baluchistan and some other Iranian provinces (37, 38). The long-eared hedgehogs like better to stay in middle climates, avoiding the hot desert and the colder mountain areas. Moreover, these species of animals prefer moderate areas with average rainfall of 100–400mm.

Regarding arid climate of Damghan City, with an average of 120mm annual rainfall, these species of mammals seem to be well adopted in the study region. However, environmental modifications induced by humans can lead to the immigration of some wild animals such as hedgehogs from their common geographical zones to humans' inhabited areas and subsequently being infected with new pathogens in new habitat (39). Similar modifications increasingly occur recently in the study area.

*Leishmania* parasites were observed in 53.3 % (8/15) of the examined animals. In microscopic positive samples, the parasites were seen in at least three of five examined organs. All the infected animals were positive for *Leishmania* species in ear lobes and hind feet smears. Using the Semi-Nested PCR method, 40% (6/15) of the samples were identified as *L. major*. Molecular methods have already been used as useful and sensitive methods for identifying *Leishmania* parasites in potential reservoir hosts without the need to parasites isolation and culture (40). *Leishmania major* parasite has also been identified and reported in one of the three (33.3 %) examined *H. auritus* from Turkmen Sahra, Golestan Province, a northern neighboring of the Semnan Province, using molecular and conventional methods (21). Natural infection of hedgehogs with *Leishmania* parasites have also been demonstrated in some other countries of the world such as Tunisia and an endemic area of ZCL in Algeria. The examined hedgehogs have been identified as *Atelerix algirus*, infected with *L. major* and *L. infantum*, in Tunisia and both *Paraechinus aethiopicus* and *Atelerix algirus*, infected with *L. major*, in Algeria (23, 24). Moreover, *Leishmania* DNA has been detected in the hair of another species of hedgehog, *Erinaceus europaneus*, in Spain (22). These findings have been suggested that hedgehogs comprise potential reservoir hosts for *Leishmania* parasites.

The parasite species were not identified in two of the eight microscopic positive samples with applied specific primers. A similar result has been found in a survey among rodents, main reservoir hosts of ZCL in the study area (11). However, some *Leishmania* species other than *L. major* may exist and circulate between sand fly vectors and animal reservoirs in the region. *Leishmania gerbilli* and *L. turanica* have already been reported in rodents from the other endemic areas of Iran (41, 42).

In the present study, all the hedgehogs were caught during three seasons; spring, summer, and autumn. The most examined animals (10/15) were caught in the summer. Five of ten (50%) animals caught in summer and all captured in autumn were shown to be infected with *Leishmania* parasites. Any animal was observed and caught in winter, possibly due to the cold weather and the animals’ hibernation state. The long-eared hedgehog spends the daytime in a burrow and may hibernate for up to 3.5 months over winter. In warmer areas, there is no extended winter hibernation but during periods of food scarcity, there may be aestivation (43). *Leishmania* species were observed and identified in the samples obtained from the hedgehogs captured in each three above mentioned seasons of the year, from May to December. The activity period of the main vector of ZCL, *Phlebotomus papatasi*, in Iran has been reported from early Apr to mid-Oct (44). In Damghan district, two peaks of activity, in Jun and Sep, have been reported for sand flies, but most of the sand fly infection has been occurred in Sep (29). Regarding the Leishmania infection of two examined animals (H1 and H2) captured in May, the animals were probably infected in the last peaks of sand flies activity and they have acted as the “maintenance hosts” of *Leishmania* parasites in the region.

The nests of captured animals were quite close to the rodents barrows. This condition could prepare a good chance of being bitten the hedgehogs by infected sand flies in resting and activity place of the animals. The presence of rodents (*Nesokia indica*) and sand flies infected with *L. major* have been demonstrated in the studied locations (11).

None of the examined mammals were shown to have any apparent skin lesions. Other studies on hedgehogs (23) and rodents as reservoir hosts of *Leishmania* species have shown similar results in a different focus of ZCL. *Leishmania major* may create no or just slight skin lesions in rodents. Therefore, it is not always simple to recognize the Leishmania infection in wild animal reservoirs such as rodents (11, 17).

Regarding the infection of the *H. auritus* with *L. major* parasites in three studied seasons (late spring to late autumn), they can be incriminated as potential reservoir hosts of the *Leishmania* spp. particularly in endemic areas of ZCL. Moreover, they can be considered as a “maintenance hosts” or even “amplifier hosts”. The “maintenance hosts” were defined as mammals infected and maintain the infection and the “amplifier hosts” were defined as the mammals that can display a characteristic that favors transmission the infection in addition to maintaining it (45). Therefore, to confirm these characteristics, more field and also experimental studies are needed in the study area.

## Conclusion

The present study demonstrated the natural infection of *H. auritus* with *L. major* parasites for the first time in Damghan City, an endemic area of ZCL in Iran. Close contact between infected rodents mainly *Nesokia indica*, hedgehogs, sensitive human population and also the presence of suitable sand fly vectors, principally *Phlebotomus papatasi*, can lead to easily exposure of *H. auritus*, a potential reservoir of ZCL, in the transmission cycle of the disease in the studied area. Besides performing the fighting programs against rodents as the main reservoir hosts of the disease in endemic areas of ZCL, infection of other susceptible mammals with *Leishmania* parasites and possibly reservoir alteration must be seriously paid attention.
